# Drone–Robot to Clean Power Line Insulators

**DOI:** 10.3390/s23125529

**Published:** 2023-06-13

**Authors:** Rogério Sales Gonçalves, Murilo De Oliveira, Murilo Rocioli, Frederico Souza, Carlos Gallo, Daniel Sudbrack, Paulo Trautmann, Bruno Clasen, Rafael Homma

**Affiliations:** 1 School of Mechanical Engineering, Federal University of Uberlândia, Uberlândia 38.400-902, Brazil; 2 CELESC, Florianópolis 88.034-900, Brazil

**Keywords:** UAVs, drones, inspection and maintenance, mobile robots, insulators

## Abstract

The inspection and maintenance of transmission systems are necessary for their proper functioning. In this way, among the line’s critical points are the insulator chains, which are responsible for providing insulation between conductors and structures. The accumulation of pollutants on the insulator surface can cause failures in the power system, leading to power supply interruptions. Currently, the cleaning of insulator chains is performed manually by operators who climb towers and use cloths, high-pressure washers, or even helicopters. The use of robots and drones is also under study, presenting challenges to be overcome. This paper presents the development of a drone–robot for cleaning insulator chains. The drone–robot was designed to identify insulators by camera and perform cleaning through a robotic module. This module is attached to the drone and carries a battery-powered portable washer, a reservoir for demineralized water, a depth camera, and an electronic control system. This paper includes a literature review on the state of the art related to strategies used for cleaning insulator chains. Based on this review, the justification for the construction of the proposed system is presented. The methodology used in the development of the drone–robot is then described. The system was validated in a controlled environment and in field experimental tests, with the ensuing discussions and conclusions formulated, along with suggestions for future work.

## 1. Introduction

The inspection and maintenance of high-voltage lines represent some of the most significant and economically costly activities for electricity supply companies. These activities need to be performed periodically, and possible failures can lead to economic, material, and human life losses. A transmission system component that requires periodic inspection and maintenance are the insulator chains. The insulator chains of high-voltage transmission lines are typically made of porcelain, glass, or composite materials (polymer insulators).

For the insulation of high-voltage lines from towers, devices known as insulators are needed. The insulators should present standardized dielectric and mechanical characteristics, considering the severe nature of the work they will perform. Insulators get dirty due to the effects of dust, salt, bird droppings, and chemicals from various smokes [1]. These dirt deposits form a conductive layer on the insulators’ surface that can cause the phenomenon of flashover. Most flashovers are unpredictable, requiring maintenance operations that take hours to complete. Dusty insulators in a transmission line can create a conductive path between the transmission line and the surrounding environment that can cause power outages and fires [1]. Therefore, cleaning the insulators is essential to prevent failures in the electrical power transmission system.

The traditional cleaning of insulator chains is usually performed by operators on live lines (termed “hot-line washing”), or when these are switched off. This cleaning can be performed by technicians using cloths or by high-pressure washers carried by the technicians. Cleaning can also be performed using trucks associated with platforms or trucks associated with robotic devices, in the case of low voltages (in the distribution system) in applications within cities. In the case of high-voltage lines in the transmission system, helicopters are also used.

The cleaning of insulators poses risks to electricians, such as falling, bodily injury, electric shock, cuts from damaged insulators, limb crushing injury, collision of the equipment with the structure and/or accessories, and bodily injury from falling tools. The average time for this task is 1.5 h per insulator chain [2].

These insulator cleaning processes can be extremely dangerous due to the high voltage passing through the transmission lines (reaching up to 500 kV), and any type of accident could be fatal, such as helicopter crashes due to electrical arcs or human errors. Aiming to reduce the risk of accidents with live line operators, various solutions have been proposed, such as the use of robots and Unmanned Aerial Vehicles (UAVs) [3,4,5,6,7], also referred to as drones in this paper.

The use of drones aims to replicate, on a small scale, the use of helicopters. Helicopters possess an advantage in terms of their larger liquid storage capacity and more powerful pumps for cleaning insulator chains, not to mention their longer flight autonomy. However, helicopter washing carries numerous drawbacks: it relies on trained operators who can accurately direct the water jet towards the object to be cleaned; the service involves costs that may make it more expensive, such as worker’s compensation insurance, fuel, the helicopter cost itself, and operators’ salaries; it demands two operators, one being a certified pilot who receives a high salary, and the other being a washer operator; and there is a significant time cost associated with helicopter takeoff and landing, in addition to the risk of accidents leading to the loss of human lives.

Conversely, the drone cleaning method would eliminate accident risks for operators; it boasts excellent geometric features that facilitate flight while navigating around obstacles, easily reaching the washing point. If connected to the ground, its water and power supply could be virtually unlimited. Without the cable, flight freedom is vast, enabling access to hard-to-reach areas. The drawback is the limited flight autonomy, as one of its greatest limitations is its battery capacity. The water supply is also reduced due to the drone’s limited payload capacity. However, refueling can be performed quickly. With the increased usage of drones, especially in crop spraying, the cost of these devices has been decreasing, making them viable for use in inspections and the maintenance of the power electrical system as well. Despite the possibility of using drones for cleaning insulator chains, there are still open issues for practical application, such as electromagnetic interferences and proving cleaning effectiveness.

This paper is organized as follows: It first provides a state-of-the-art review of insulator chain cleaning, followed by a description of the proposed drone–robot solution. Afterward, the experimental setup is detailed, followed by a presentation of the experimental results. Finally, the paper concludes by summarizing the achieved results.

### Review of the State-of-the-Art in Cleaning Insulator Chains

State-of-the-art solutions for insulator chain cleaning aim to automate the cleaning process, utilizing robots, drones, or helicopters. The cleaning of insulator chains with robots generally follows the same format, using complex devices that move along or around the insulator disks to perform mechanical cleaning [8,9,10,11,12]. The major challenge in using these robots lies in maintaining complex prototypes and placing them on the insulators, which can be risky as technicians must climb and place the robots on the insulators. Ergonomic issues also arise due to the robots’ heavy mass. Different sizes and types of insulators further complicate the adoption of a mechanical design that suits all. It is noteworthy that the routine placement of these robots on insulator chains by technicians requires the transmission system to be shut down on that specific line.

The cleaning of Insulators using a helicopter with an attached high-pressure washer was initially described by Kurtgis in 1984 and is widely used today [4,5]. Besides the high cost associated with this operation, there is always the possibility of accidents resulting in the loss of human lives.

In the patent [13], a drone with three nozzles for cleaning insulators is presented. One nozzle is for the cleaning product, another is for clean water, and a third is for the high-pressure spray. The cleaning process is monitored by a camera, with the images sent to a ground station where the operator determines whether the cleaning has been completed. This patent presents a promising idea but lacks detail, further development, and a prototype.

The company Aerones developed a drone that could be applied to insulator cleaning. This drone has a load capacity of 200 kg and is powered by electricity and water through cables connected to a ground unit [5]. In addition, there is the work developed by [14], who presents the idea of using a helicopter or drone model to perform insulator cleaning using a water source such as a truck tank coupled to the drone by a hose. When physically connecting the drone to the water and energy source on the ground through cables, cleaning should occur with a water mist to avoid a continuous jet that could cause a potential differential, damaging the equipment and possibly leading to accidents on the ground with workers. The cable connection also limits the drone’s maneuverability during flight.

In [15], a prototype of a small drone for localized pollutant cleaning using a vision system to identify the dirty area and clean it was introduced. Simulated tests were conducted, presenting an interesting solution, but it lacked validation in a real environment with energized lines.

It is also noteworthy that the solutions presented in the literature do not provide scientific backing regarding the design and its effectiveness, nor do they present the necessary requirements for practical cleaning. To design the drone–robot proposed in this paper, normative recommendations were considered, such as the IEEE Guide for Maintenance Methods on Energized Power Lines, Std 957-2005 [16], which deals with safe and suitable procedures for cleaning insulators using various methods. Thus, the cleaning procedures of the proposed drone–robot will be adjusted to the recommendations in this standard.

The Standard provides the types of cleaning traditionally employed, with water washing being the primary cleaning method using different pressures: high pressure (400 psi to 1000 psi); medium pressure (300 psi to 400 psi) and low pressure (200 psi). Cleaning with deionized water is recommended for porcelain and glass insulator chains. The frequency of cleaning varies depending upon the degree of contamination, the weather conditions, and the particular insulator design.

Fog, mist, or light rain usually create conditions that produce a conductive film on the dirty insulator surface without washing the impurities off the surface. This conductive film can lead to problems and interrupt the energy supply. Different contaminants have been identified and are a function of local agricultural, industrial, and geographic conditions. These contaminants include salt, cement, dust, bird excrement, chemicals, smog (from automobile emissions), cooling tower effluents, and smoke. Table 1 presents the main contaminants found and what can be cleaned with pressurized water.

Salt contamination mainly affects coastal areas, leading to a high concentration of salt deposits on the insulator surface. Salt will quickly dissolve and wash off in heavy rain or a stream of water.

Table 1 shows that the main contaminants can be removed using deionized water with low- and high-pressure jets. The water expelled by the washer should be in a spray format to avoid interference between the insulator chain and the drone.

The primary contribution of this paper is the design of a drone–robot, with the project detailed for practical application in cleaning energized insulator chains. The drone–robot proposed for cleaning insulator chains was conceived as a device aimed at minimizing the possibility of accidents involving live line technicians who carry out the inspection/maintenance of high-voltage cables. It also seeks to reduce the occurrence of failures due to interruptions in the electric power supply, damage to industrial and residential consumer equipment, and maintenance costs, thereby increasing the energy system’s efficiency. Current work developing insulator chain cleaning solutions is based on initial ideas without proven effectiveness. Cleaning methods using water supplied to a drone with a ground reservoir pose a short-circuit risk if the water jet from washing is continuous. The solution presented in this paper uses a drone–robot with all necessary onboard devices and takes advantage of the electrical insulation provided by atmospheric air, acting as a dielectric to prevent unwanted electrical discharges.

This paper presents a new robotic module that solves the problem of cleaning insulator chains based on an integrated project between the robotic module and drone with an innovative algorithm, using a depth camera capable of guiding the safe cleaning distance. For the implementation of this drone–robot for cleaning the chains of insulators, several problems had to be considered, such as the effective cleaning distance for the chain, safety mechanisms to avoid collision between the equipment and the chain of insulators, and the calculation of the shielding of the control system against interference from the energized chain of insulators.

In summary, the innovations proposed with the drone–robot for cleaning insulator chains are as follows: the creation of an innovative maintenance solution that aims to produce a safe insulator chain washing procedure that is mathematically and scientifically grounded. Unlike other solutions, it features the semi-automated sensing of the cleaning operation. The operator’s role is to position the drone relative to the chains, assisted by a monitoring system that measures the drone’s distance from the insulator chain using a depth camera. The presentation of simulated experimental tests shows the cleaning procedure and its effectiveness, and experimental tests in a real scenario demonstrate the solution’s effectiveness against electromagnetic interferences.

## 2. Materials and Methods

**Figure 1:**
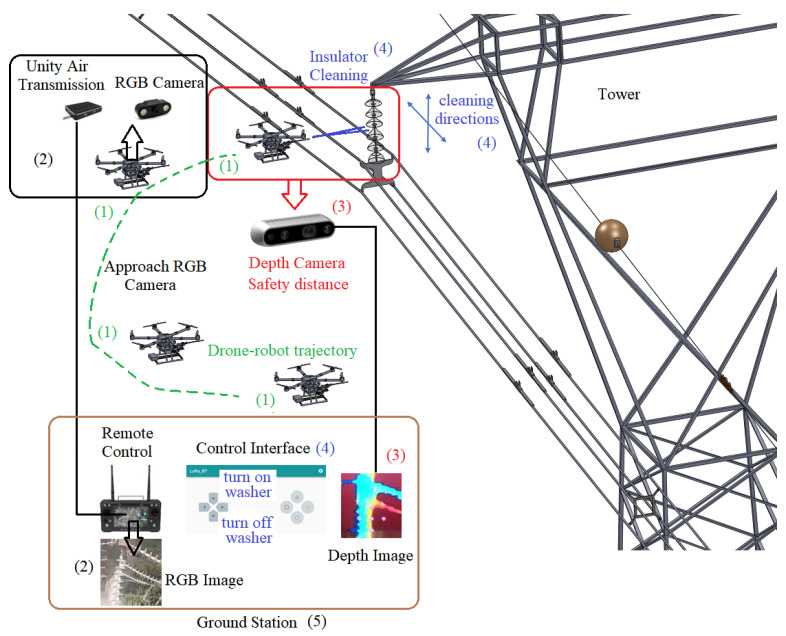
Conceptual design of the proposed solution. (1) The drone–robot’s approach is carried out by manual commands sent by an operator (Drone–robot trajectory); (2) To facilitate the approach, an RGB camera attached to the drone is used, transmitting the images to the ground station (5). The fine approach to the insulator chain is performed by a depth camera (3). When the drone is in a suitable region for cleaning, the washer (4) is activated from the ground station (5). The movements to clean the chain are manually performed (4).

The main goal of this paper is to develop a drone–robot system for insulator cleaning on 69 kV and 138 kV power lines, as shown in Figure 1. Firstly, the system positions itself near the insulator chain using an RGB camera on the drone, which transmits the image in real-time to the drone remote control at the ground station. The drone flight is conducted by one operator in this paper. Then, a vision algorithm from a depth camera provides the distance of the drone from the insulator chain, with indications of a safe distance between the drone–robot and the insulator chain. If the distance is within the safe cleaning range, the pressure system is activated, and the drone operator starts to perform the movements (up and down and sideways) to clean the insulator chain, Figure 1. Checking whether the cleaning was successful is performed by acquiring insulator chain images using a specific drone for inspection.

### 2.1. Drone Description

**Figure 2:**
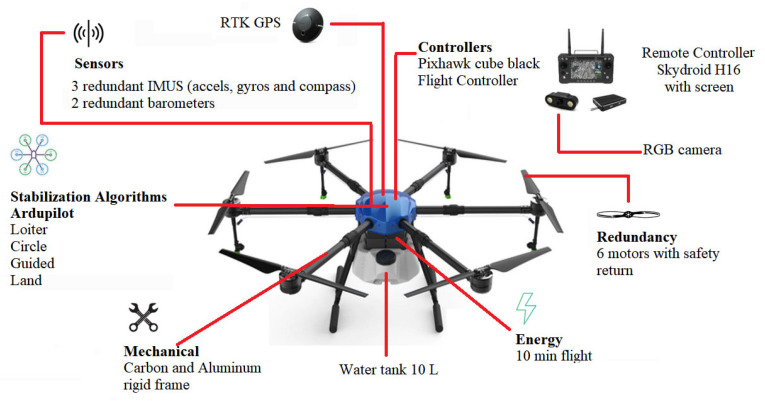
Hexacopter configured from the EFT E610 frame.

This project used the structure of a hexa from the EFT company, model E610. It was designed for use in agriculture for sprays, containing a reservoir with a capacity of 10 L. The drone assembly used six DC Hobbywing XRotor Pro X6 motors, six 80A ESCs (Electronic Speed Controllers from Hobbywing, Shenzhen, China), a Pixhawk 2.1 Cube Black flight controller, and RTK GPS. Together with this, a Skydroid H16 radio control was used. The total takeoff weight was 25 kg. Figure 2 shows the configured drone’s main characteristics.

The drone controller firmware used was Ardupilot [17]. The user interface with Ardupilot was handled through the Mission Planner software [18], where the drone’s configuration and calibrations are carried out. It is possible to visualize the flight characteristics in real time, as well as the alterations in all parameters related to the controller configuration. In this paper, the Loiter flight mode was used as a basis, in which the drone remains hovering at a fixed point in space. After any manual movement, the drone reestablishes itself, hovering at a fixed position in the air.

The drone’s positioning relative to the insulator chain is carried out with the aid of a dedicated RGB camera positioned on the drone, the images of which are sent to the radio control at the ground station, as shown in Figure 1 and Figure 2. In this way, the drone’s flight is carried out manually. To assist in maintaining the necessary distance for cleaning the insulator chains, a vision system made up of a depth camera was used, as shown in Figure 1.

### 2.2. Robotic Module for Cleaning the Insulator Chain

**Figure 3:**
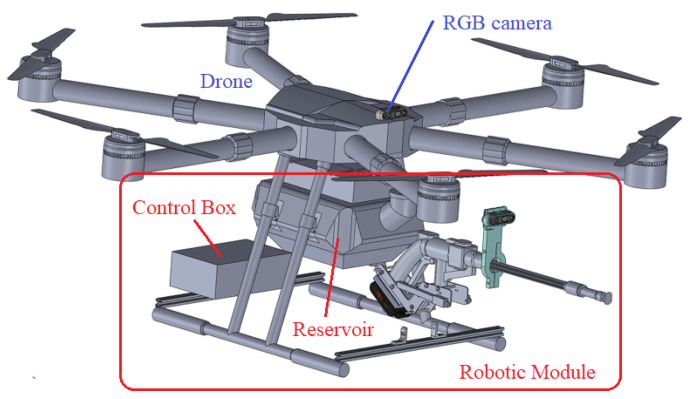
Drone–robot for cleaning insulator chains.

**Figure 4:**
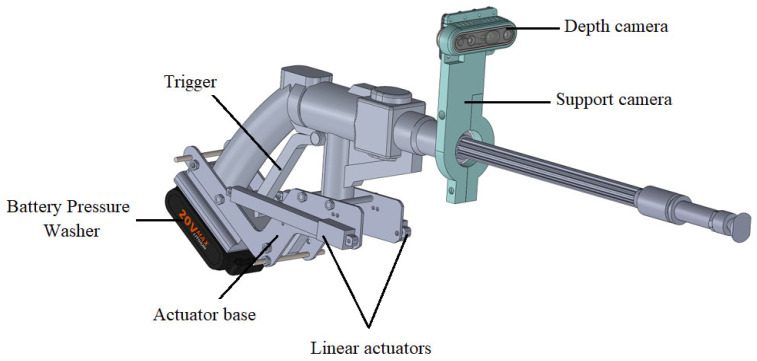
Robotic module for washing insulator chains.

Figure 3 and Figure 4 show the robotic module for cleaning the insulator chain. It is composed of a portable battery-powered washer, a trigger actuation system for the washer, a depth camera, and a control system.

The washer was selected based on the cleaning requirements for insulator chains outlined in Std 957-2005. The washer is a WORX brand with a pressure of 320 psi, thus considered a medium-pressure washer, and suitable for cleaning the main contaminants as per Table 1. It is powered by a 20-volt battery. Experimental tests that were carried out determined the washer’s flow rate to be 1.6 L/min. The washer’s water supply was directly connected to the drone’s water reservoir. The water must be demineralized to avoid conducting electricity, and the water jet must be in the form of droplets/spray, that is, not continuous.

The system for activating the washer’s trigger was developed by measuring the force required to operate it using a load cell, obtaining a result close to 2 kg. Thus, two linear actuators were used, each with a stroke of 100 mm, a speed of 30 mm/s, a 12 V power supply, and a force of 25 N each, as shown in Figure 4. The trigger activation is carried out from the ground control unit.

A depth camera was used to detect the insulator chain and maintain a safe distance between the insulator chain and the drone, as shown in Figure 4. The depth camera used was the Intel RealSense D435, specially designed for use in drones. It has a nominal measurement range of 20 mm to 4000 mm with a resolution of 1 mm. The control system is detailed in Section 2.4.

### 2.3. Development of Electromagnetic and Electrostatic Shielding for the Drone–Robot

**Figure 5:**
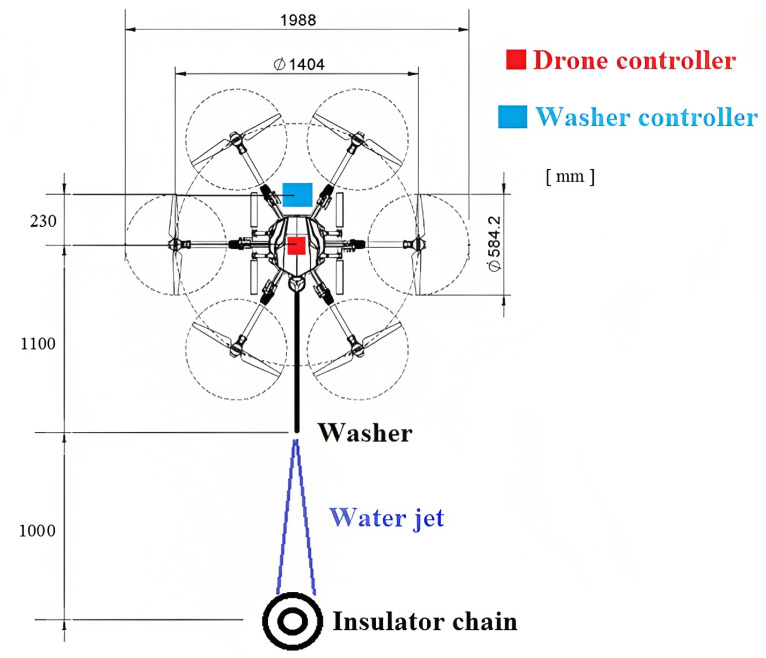
Cleaning drone–robot scheme top view [mm].

The cleaning drone–robot was designed for cleaning insulator chains on cables that carry voltages of 69 kV or 138 kV. Given the potential for intense electromagnetic interference and undesired electrical discharges to damage the drone–robot, shielding against electromagnetic interference and electrostatic discharges was designed and implemented. The distances considered for calculations are presented in Figure 5.

To shield the robotic module’s electronic system (washer controller, Figure 5), a prismatic box was used so that the electronic components could be inserted inside it, as shown in Figure 3. The box’s thickness needed to be determined to make it effective for shielding. The design of the shielding for the drone’s control system (Figure 5) was also carried out. For this purpose, the practical procedure proposed by [19] was followed and adapted to the robot–drone application.

In source [20], it was identified that electromagnetic emissions from transmission lines occur in three specific frequency bands: 30 to 3000 Hz, which is related to the AC voltage frequency found in 50 to 60 Hz transmission lines and their respective harmonics; 0.15 to 3 MHz, created by corona discharges, which can be seen in both cables and insulator chains; and 30 to 300 MHz, which stems from the field produced by the corona effect as well.

Research [21] inspected the corona effect’s presence in 138 kV line insulators by using ultraviolet cameras. Normally, there was no significant corona effect noticed, only infrequent sparks that were considered negligible. As a result, the frequencies’ effects in the second and third emission ranges can be disregarded.

However, one can consider a critical situation in which Partial Discharges (PD) occur in the insulator chain whose emitted electric field exceeds the dielectric strength of air. The effect of the designed shielding under this critical condition was also evaluated.

For the shielding design, the attenuation calculation approach suggested by [19] was used to examine the shielding impact in the frequency band of 30 to 3000 Hz, and for partial discharge phenomena.

Initially, it was proposed that the drone control system’s shielding would be carried out with 0.1 mm thick aluminum sheets, and the module’s electronic system shielding would be performed with a prismatic box 0.7 mm thick. If the proposed 0.1 mm thick shielding is sufficient to attenuate the interferences, the shielding for the module control system can also be considered designed, as it will show greater efficiency due to the thicker aluminum and greater distance from the interference emitting source.

**Figure 6:**
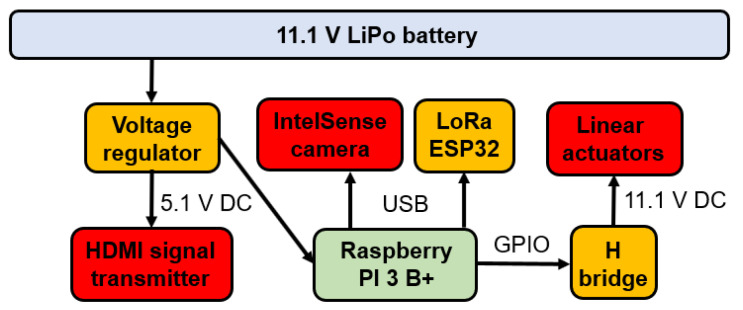
Scheme of the module electronic system for cleaning insulator chains.

The initial set of data necessary for the calculations involved in this procedure is as follows:

- v = 3 × 10^8^ m/s (speed of electromagnetic waves);
- r = 2.1 m (minimum distance of the source at which the drone controller will be exposed), Figure 6;
- μ_r_ = 1 (relative magnetic permeability of aluminum);
- σ_r_ = 0.61 (relative conductivity of aluminum);
- t = 0.1 mm = 0.0039 in (shield thickness).

To ascertain whether the field that impinges on the shielding is near or far, Equation (1) is utilized, taking into account the minimum and maximum frequencies that need evaluating, which are 30 Hz and 3000 Hz. For the field to be considered near, the following criteria must be satisfied for the two frequency boundaries:

(1) $$\frac{\lambda }{2\pi }=\frac{v}{2\pi f}\;\geq \;r$$

The minimum frequency yields 1,591,550 > 2.1, while the maximum frequency produces 15,915 > 2.1. Based on Equation (1), it is evident that the system encounters near-field emissions. As a result, it is crucial to address both electric field and magnetic field interferences separately.

Equation (2) is used to examine the reflection and absorption effects, where S stands for the shielding effectiveness, A represents the absorption effect, R symbolizes the reflection effect, and B is a correction factor associated with the influence of thin shielding walls.

(2) S=A+R+B [dB]

The absorption effect (A_E_) calculation is given by Equation (3), with t representing the shielding thickness in inches.

(3) $$A_{E}=3.34\;t\;\sqrt{{f\mu }_{r}\sigma_{r}}$$

The reflection effect for electric fields (R_E_) is calculated by Equation (4), with r being in meters:

(4) $$R_{E}=322+10\log\left(\frac{\sigma_{r}}{\mu_{r}f^{3}r^{2}}\right)\;$$

The B factor for electric fields (B_E_) can be dismissed:

(5) $$B_{E}=0$$

Even though Equations (3) and (4) involve the magnetic permeability of the shielding medium as a variable, they are indicated for calculating the attenuation of the electric field effect, according to the method proposed in [19]. This occurs because Equation (3) is derived from the skin depth (δ) equation, which is the thickness at which the wave intensity is approximately 37% of its original value when it penetrates the shielding material (Equation (10)). Upon replacing the variables in the previous equations, the shielding effectiveness for the electric field (S_E_) can be computed as follows:

(6) $$S_{E}{=A}_{E}{+R}_{E}{+B}_{E}=0.0102\sqrt{f\;}+322+10\log\left(\frac{0.1383}{f^{3}}\right)$$

To estimate the magnetic field attenuation, the calculation for the absorption effect (A_M_) is the same as in the case of the electric field computation, using Equation (7):

(7) $$A_{M}=0.0102\sqrt{f\;}\;$$

The reflection effect (R_M_) is calculated by Equation (8), with r being in meters:

(8) $$R_{M}=14.6+10\log\left(\frac{{\mathrm{fr}}^{2}\sigma_{r}}{\mu_{r}}\right)$$

To calculate the B_M_ factor, Equation (9) is used, where δ denotes the “skin depth”, which is the thickness at which the wave intensity is approximately 37% of its original value, measured in inches:

(9) $$B_{M}=20\log\left({1\;-\;e}^{-2t/\delta }\right)$$

The computation of δ for aluminum is performed using Equation (10), where μ_r_ is the magnetic permeability of aluminum, and σ_r_ is the relative conductivity of aluminum:

(10) $$\delta =\frac{2.6}{\sqrt{{f\mu }_{r}\sigma_{r}}}$$

The magnetic field final effectiveness is determined by Equation (11):

(11) $$S_{M}{=A}_{M}{+R}_{M}{+B}_{M}=0.0102\sqrt{f\;}+14.6+10\log(2.69f)+20\log(1\;-\;e^{-0.0023\sqrt{f}})$$

Equations (12) and (13) are applied to calculate the attenuated fields, with the effectiveness values of S_E_ and S_M_ established:

(12) $$S_{E}{=20\;\log\;(E}_{0}{/E}_{f})$$

Here, E_0_ is the incident electric field, and E_f_ is the attenuated electric field.

(13) $$S_{M}{=20\;\log\;(H}_{0}{/H}_{f})$$

Similarly, H_0_ is the incident magnetic field, and H_f_ is the attenuated magnetic field.

The computations for attenuation apply to the frequency range of 30 Hz to 3000 Hz (with a shielding thickness of t = 0.1 mm). The minimum effectiveness found was 209.658 dB at a frequency of 3000 Hz. The attenuation multiplier for this frequency range is extracted from Equation (12):

(14) $$20\;\log\;\left(E_{0}{/E}_{f}\right)=209.658$$

(15) $$E_{f}=3.289\cdot 10^{-11}\cdot E_{0}$$

The maximum electric field given off by a 220 kV transmission line was calculated to be 19 kV/m (190 V/cm) in normal operation conditions, as estimated by research [22]. It follows that if the shielding can curb the field created by a 220 kV line, it can likewise attenuate the field generated by a 138 kV line, as its amplitude will be less. With a maximum value of 190 V/cm serving as the electric field emitted by a 220 kV line with a fundamental 60 Hz frequency [23], the designed shielding effectiveness can be assessed. The electric field incident within the internal circuit of the shielded structure can be determined by utilizing Equation (16):

(16) $$E_{f}=3.289\cdot 10^{-11}\cdot 190\;V/\mathrm{cm}=6.250\;\mathrm{nV}/\mathrm{cm}$$

The insignificance of an electric field of an nV/cm order of magnitude can be dismissed concerning the induction of unwanted voltages in circuits supplied at 5 V to 11.1 V, which is the current case. Given that the shielding satisfies the minimum effectiveness needed to guard against the current electric field’s highest amplitude, it is expected to be more successful in diminishing electric fields of various frequencies within the evaluated range.

It is suggested that in a dry environment, the frequency of the emitted electric field from gap discharges can reach up to 6 GHz [24]. In gap discharges, air’s dielectric breakdown is a complete, rather than partial, breakdown. Regarding the interference emitted by fields generated by partial discharges (PD), due to the lack of information about the frequency of the emitted fields, this maximum frequency value for gap discharges will be assumed in the attenuation calculations for the electric field from partial discharges.

In the frequency range of 300 MHz (above the maximum frequency emitted by the corona effect, as stated in [20]) and 6 GHz, the lowest shielding effectiveness (S_E_) found was of 258.967 dB (at 300 MHz frequency). Applying Equation (12) to determine the attenuation multiplier for this case yields the following:

(17) $$20\;\log\;\left(E_{0}{/E}_{f}\right)=258.967\;\mathrm{dB}$$

(18) $$E_{f}=1.126\cdot 10^{-13}\cdot E_{0}$$

Considering the electric field value as the one required for the partial breakdown of the dielectric strength of air, which is 30 kV/cm (30,000 V/cm) [25], the electric field after attenuation is determined according to Equation (19):

(19) $$E_{f}=1.126\cdot 10^{-13}\cdot 30000\;V/\mathrm{cm}=3.379\;\mathrm{nV}/\mathrm{cm}$$

As with the previously obtained attenuation, the attenuated electric field here is in the order of nV/cm, which can be neglected.

To attenuate the interference from magnetic fields generated by partial discharges (in the range of 300 MHz to 6 GHz), the lowest effectiveness found using Equation (11) was 258.967 dB (at the frequency of 300 MHz). This high attenuation value proves to be sufficient for shielding against magnetic fields, similar to the shielding obtained for the evaluated electric fields.

Considering the magnetic field emitted in the range of 30 to 3000 Hz, a structure with a thickness of 0.1 mm fails to provide adequate shielding against magnetic fields, using Equation (11). Then, the interference caused by magnetic field emissions of a 60 Hz frequency was inspected by employing an experimental setup that included a microwave oven, a gaussmeter, and an electronic circuitry similar to that used in the insulator cleaning drone robot, which was encased within a 1 mm thick aluminum enclosure. The emitted magnetic field was measured using the TAMB-3D gaussmeter from Globalmag, which has a 0.001 mT resolution and a 5 mT nominal range.

A magnetic field at 60 Hz of approximately 15 μT at the center is emitted by 138 kV 12-phase power lines, as per [23]. This magnetic field value serves as a reference value. If the electronic circuitry can operate normally under magnetic fields of this magnitude or higher, then no magnetic field shielding is needed for this frequency range.

During the magnetic field emission, the circuit showed no abnormal functioning when exposed to a maximum magnetic field of 200 µT. This could either be because the magnetic fields of this amplitude are too feeble to cause interference, or due to the absence of the magnetic coupling (DC frequency and no transmission frequency via USB protocol) of undesired signals from the magnetic field to which the module was exposed.

The system’s shielding against the referenced electromagnetic interferences is confirmed with these findings, applicable to both the drone’s controller box and the module’s electronic control system.

Regarding electrostatic shielding, the energized lines closest to the drone are prevented from arcing towards it due to the dielectric properties of air. This negates the need for an electrostatic shielding design. The drone and the insulator chain’s separation at this distance is adequate to forestall discharges resulting from an air dielectric breakdown. In regard to electrostatic shielding, the dielectric properties of air prevent the closest energized lines from arcing towards the drone. Consequently, there is no need to incorporate an electrostatic shielding design into the drone. Considering a minimum distance of 1 m between the washer’s tip and the energized chains, it was decided that the drone’s structure would not be shielded, as this distance between the drone and the chain is sufficient to prevent discharges due to air dielectric breakdown, as shown in Figure 5.

### 2.4. Electronic System of the Robotic Module

The washer electronic system was developed based on an embedded Raspberry Pi 3B+ controller, responsible for the system’s logical operation and connection to peripheral devices through its I/O bus. The LoRa ESP32 communication board and IntelSense depth camera are connected to this board. The two linear actuators used to trigger the washer are connected to an H bridge, which activates both actuators simultaneously. This H bridge is triggered by a digital output commanded by the Raspberry Pi 3 B+ board. The depth camera image is transmitted via an HDMI signal transmitter, which operates with its respective receiver at the ground station. The system is powered by an 11.1 V battery, the voltage of which was lowered to 5.1 V to power the system’s components. Figure 6 presents a schematic of the described electronic system.

The electronic system was inserted into an aluminum box with a thickness of 0.7 mm, due to the electromagnetic interference calculations described in Section 2.3.

The message traffic from the ground station to the robotic module is carried out in the following way: the smartphone is connected via Bluetooth to the emitting LoRa board. The “Bluetooth Serial Controller” application is programmed to send messages to this LoRa. The emitting LoRa sends the received message to the receiving LoRa, present in the robotic module, which communicates via USB with the Raspberry Pi 3B+. The program running on the Raspberry Pi 3B+ interprets this message and performs the required action. Messages are all sent via serial communication, that is, in one way. When an application button is pressed, only one message is sent.

**Figure 7:**
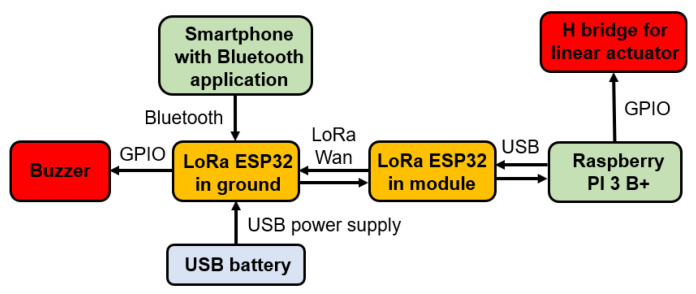
Communication system scheme present in the module.

This communication also occurs in the opposite direction: when there is a need to notify the ground operator about the drone’s position relative to the insulator chains, the message is sent from the Raspberry Pi 3B+ to the ground LoRa, passing through the LoRa present in the robotic module. A buzzer connected to the LoRa at the ground station emits sound signals according to the current drone distance configuration. Figure 7 shows a communication scheme.

**Figure 8:**
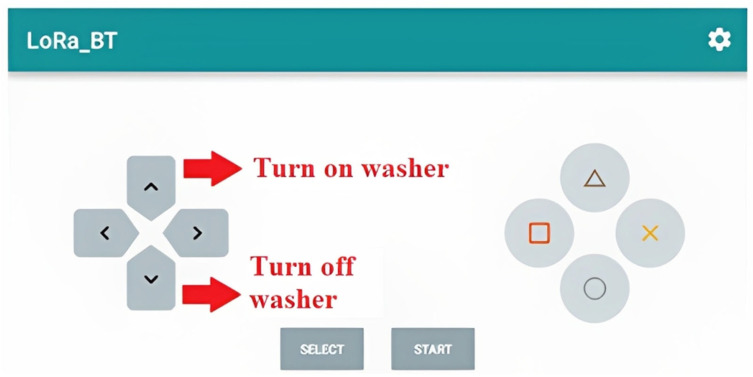
“Bluetooth Serial Controller” application control interface, with buttons configured for use highlighted.

Each button of the “Bluetooth Serial Controller” application interface was configured to perform a function. In this case, buttons were used to turn the washer on and off. Figure 8 shows the Bluetooth communication program interface, with the buttons used in the module’s operation highlighted.

Regarding the electronic system operation logic, a program is responsible for coordinating the reception of messages from the LoRa ESP 32. In this case, the only commands to be sent from the ground to the drone are the activation or deactivation of the washing gun. An auditory warning system for the ground operator was implemented using LoRa communication. Thus, communication with LoRa was configured as bidirectional. While the LoRa receives messages indicating the execution of the module’s functions, it also sends messages to the control station’s LoRa, indicating the current state through auditory signals. The LoRa at the control station was equipped with a buzzer connected to its input and output bus, which emits the auditory signal. A USB battery was connected to the ground LoRa to enable continuous operation (Figure 7). Another program pertains to the depth camera operating logic, the operation of which is described in as following.

### 2.5. Robotic Module Distance Detection System

The operation of cleaning the insulator chains requires the cleaning drone–robot to operate within a distance range of 1 m to 1.5 m from the insulator chain so that the water jet can effectively clean the target chain. While the drone is flying in Loiter mode, it is possible to enable a function in which the operator is notified of the drone’s distance from the insulator chain. An Intel RealSense D435 depth camera was used to detect the distance between the insulator chain and the drone. This depth camera is connected via USB to the Raspberry Pi, as shown in Figure 6. The images captured by the depth camera are visualized via wireless HDMI communication, with the HDMI transmitter connected to the Raspberry Pi’s HDMI output.

The depth camera was positioned on the washer’s boom at a height of 0.08 m using a 3D-printed part, as shown in Figure 4. The height was determined experimentally to capture the image centrally without interference from the washer’s tip.

**Figure 9:**
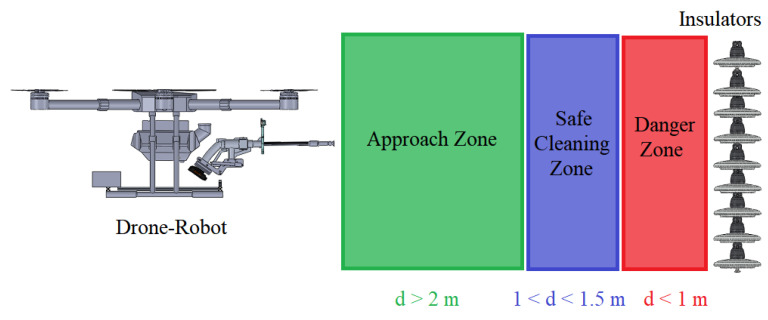
Detection scheme of the distance to the insulator chain using the depth camera.

The depth camera program developed to operate on the Raspberry Pi displays the image captured by the camera. To improve the camera’s distance detection, a point diagram was implemented, where the distance of each point is archived. Five detection points are positioned in the image center, with a central point and four other points inserted to the right, left, above, and below the central point. As the depth camera image captures some blind spots (i.e., points at which distance reading is not performed), these five points serve as redundancy in detection, and they are arranged to facilitate the detection of the insulator chain’s irregular geometry. The reading that presents the shortest distance obtained is then captured by the program. Then, an auditory signal is sent, notifying the operator about the drone’s position. As the drone–robot operator approaches the insulator chain using the drone’s RGB camera, the developed vision system calculates the chain’s distance. If the distance is less than 1.5 m and greater than 1.0 m, an intermittent sound signal is emitted to indicate to the operator that the drone is at the prescribed cleaning distance. If the distance is less than 1m, a continuous sound signal is emitted at the ground control station, and the pilot moves the drone away from the chain, repeating the approach procedure, as shown in Figure 9. Algorithm 1 presents the algorithm developed.
**Algorithm 1: Depth camera image streaming and distance processing to warn drone pilot**1:*StartCameraStream <- CameraParameters*2:**while** 
*(true)* 
**do**
3:*            DepthMatrix ← ImageDepthFrames*4:*            Distance1 ← DepthMatrix (ImagePosition1)*5:*            Distance2 ← DepthMatrix (ImagePosition2)*6:*            Distance3 ← DepthMatrix (ImagePosition3)*7:*            Distance4 ← DepthMatrix (ImagePosition4)*8:*            Distance5 ← DepthMatrix (ImagePosition5)*9:**            if** 
*(Distance1 < Distance2, 3, 4 and 5)*  
**then**
10:*                      CurrentDistance ← Distance1*11:**            else if** 
*(Distance2 < Distance1, D3, D4 and D5)*  
**then**
12:*                      CurrentDistance ← Distance2*13:**            else if** 
*(Distance3 < Distance1, D2, D4 and D5)* 
**then**
14:*                      CurrentDistance ← Distance3*15:**            else if** 
*(Distance4 < Distance1, D2, D3 and D5)*  
**then**
16:*                      CurrentDistance ← Distance4*17:**            else if** 
*(Distance5 < Distance1, D3, D4 and D5)* 
**then**
18:*                      CurrentDistance ← Distance5*19:**            if** 
*(CurrentDistance ≤ 1 m)*  
**then**
20:*                      Buzzer ← ConstantBuzz*21:**            else if** 
*(CurrentDistance ≥ 1.5 m)* 
**then**
22:*                      Buzzer ← Silence*23:**            else if** 
*(CurrentDistance > 1 m and CurrentDistance < 1.5 m)* 
**then**
24:*                      Buzzer ← IntermitentBuzz*

**Figure 10:**
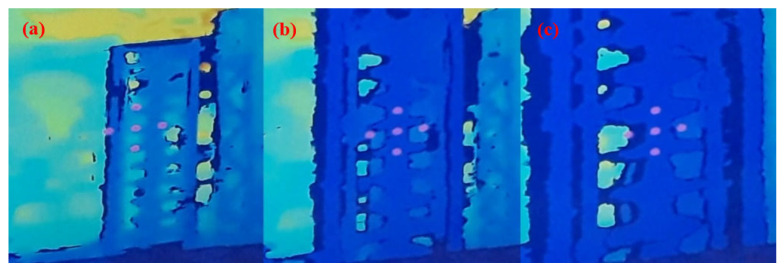
Images obtained using the depth camera, displaying the point diagram, in various situations: (**a**) off-target detection; (**b**) optimal washing distance; (**c**) very close to the chain (less than 1 m).

Figure 10 shows the program in operation, with the depth camera facing an insulator chain mounted on a metal structure on the ground, where, even in the presence of various noise sources, the algorithm worked with the necessary distance detection.

### 2.6. Drone–Robot Prototype for Cleaning Insulator Chains

**Figure 11:**
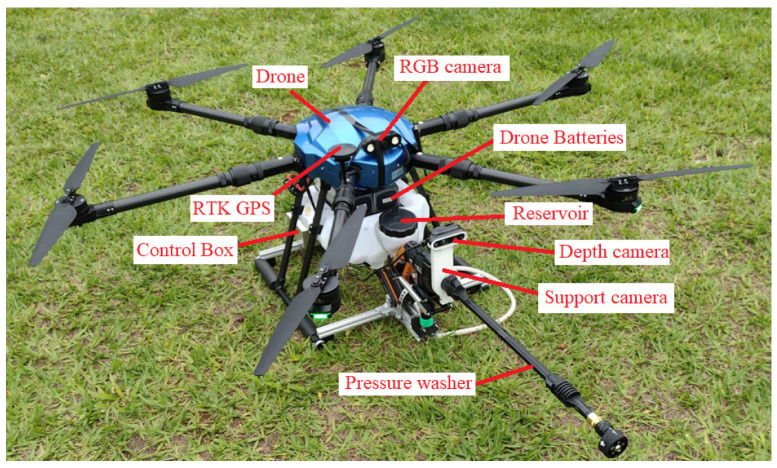
Drone–robot prototype.

The drone–robot designed for cleaning insulator chains and other elements is conceptualized as a robot to which a drone is affixed with a detachable module, enabling cleaning. The cleaning targets could be insulators, cables, or signal spheres, among others, with this study focusing on cleaning insulator chains. Figure 11 shows the drone–robot prototype, highlighting its main components. The complete robotic module weighs 3.5 kg.

This robotic module has its structure fixed to the drone during operation. It features hybrid functionality, encompassing both radio-controlled and semi-automated functions. Among these, the semi-automated functions are maintaining a safe distance from the cleaning target and the washer’s activation via ground command.

**Figure 12:**
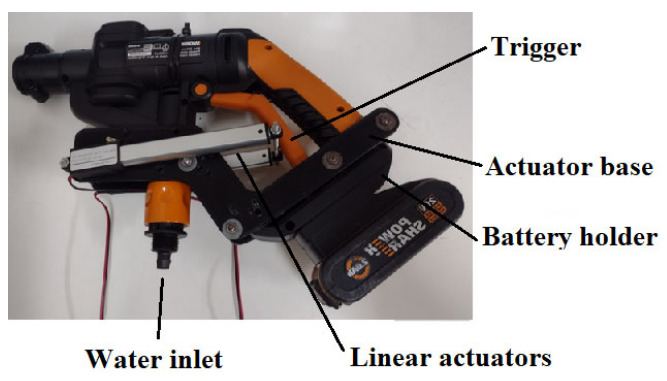
The cleaning module’s washing gun with corresponding components.

Aluminum profiles were attached to the drone’s landing gear to secure both the box containing the robotic module control system and the washer. A base for mounting the linear actuators on the washer was 3D printed, as shown in Figure 12.

The designed drone has a total takeoff capacity of 25 kg. Considering the robotic module’s mass of 3.5 kg and the mass of other elements, such as the frame, batteries, etc., the robotic module has a water storage capacity of 8 L.

Practical tests carried out in the Laboratory of Automation and Robotics (LAR) at the Federal University of Uberlândia evaluated the washer’s flow rate to be 1.6 L/min. This enables two tank empties per drone battery charge during cleaning operations, i.e., during the drone’s estimated 10 min airtime.

The appropriate cleaning distance for the insulators was verified via a test in a high-wind environment, between position 4 and 5 on the Beaufort force scale (approximately 8 m/s). The distance measurements between the washer’s tip and a pole simulating the insulator chain to be cleaned were taken with a simple tape measure. Following the tests, it was concluded that washing should be performed 1.5 m away to impact the insulators with considerable force and at most 1 m away to prevent possible drone damage and electric discharges, according to the calculations from Section 2.3.

Table 2 summarizes the specifications related to the cleaning of the insulator chains defined for the drone–robot project.

## 3. Results

### 3.1. Simulated Cleaning Tests

**Figure 13:**
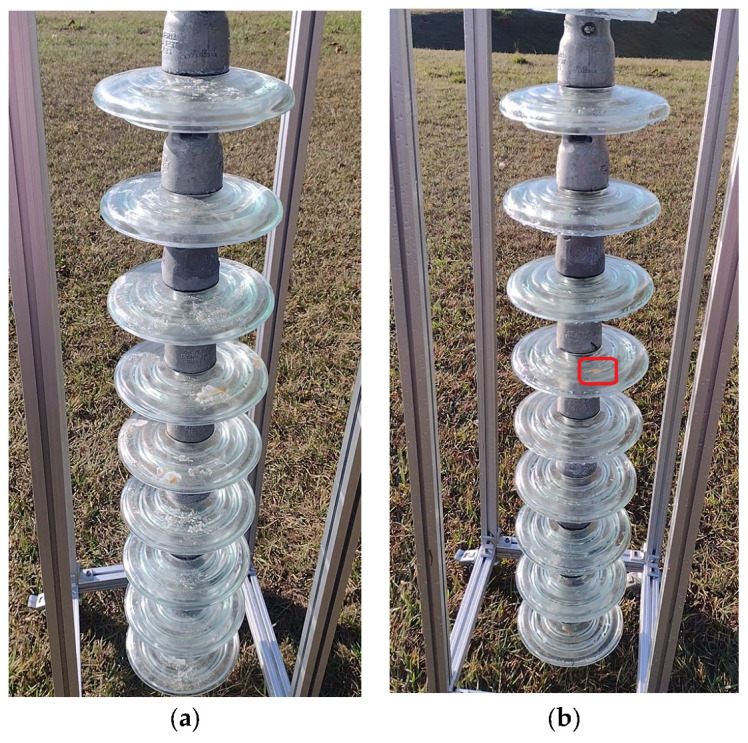
Insulator chain constructed for tests: (**a**) insulators covered with a 35% saline solution; (**b**) cleaned insulator chain. Front perspective.

For the execution of the simulated experimental tests, a support for the insulator chains was made using aluminum profiles so that they were arranged vertically, as shown in Figure 13. The chain used in the tests contained 10 insulators. The insulators used had a diameter of 255 mm and a pitch (height) of 146 mm. These were to be used on 69 kV (5 discs) and 138 kV (9 or 10 discs) lines.

**Figure 14:**
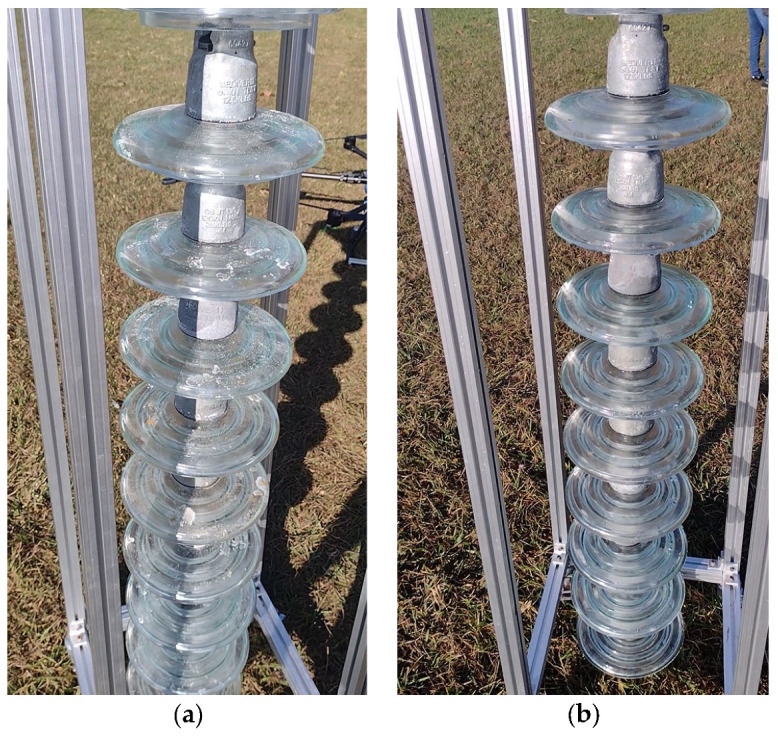
Insulator chain constructed for tests: (**a**) insulators covered with a 35% saline solution; (**b**) cleaned insulator chain. Left-side perspective.

**Figure 15:**
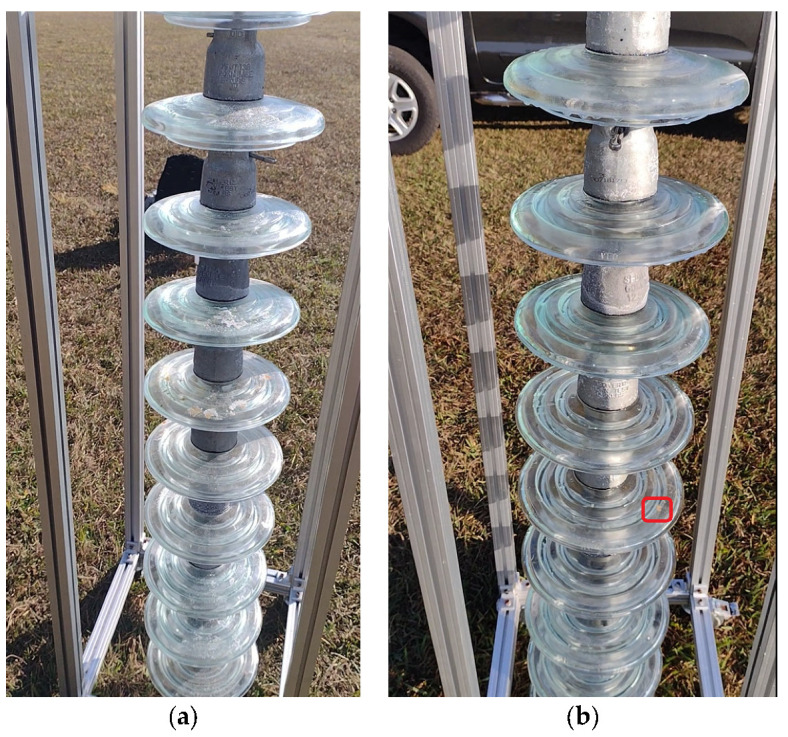
Insulator chain constructed for tests: (**a**) insulators covered with a 35% saline solution; (**b**) cleaned insulator chain. Back perspective.

**Figure 16:**
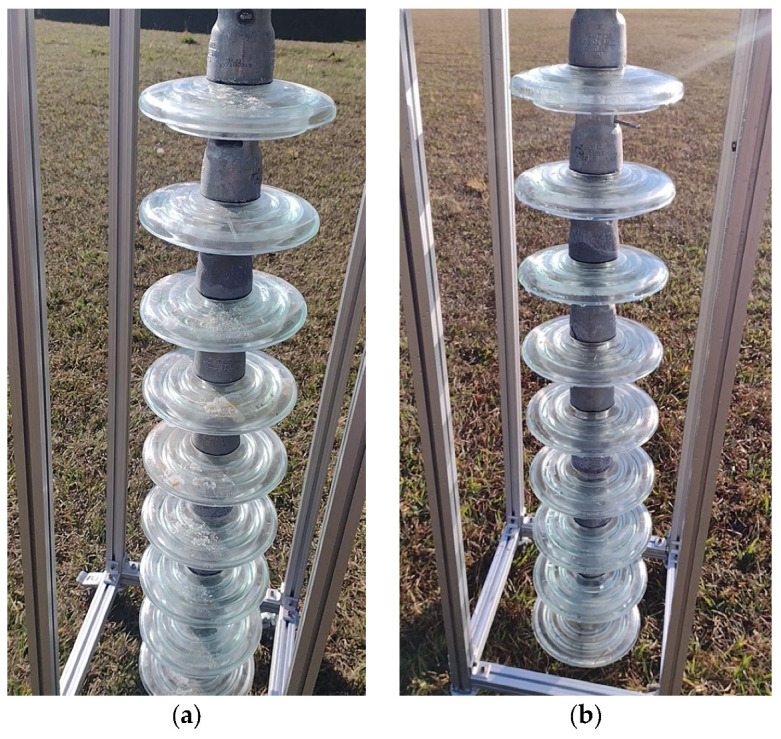
Insulator chain constructed for tests; (**a**) insulators covered with a 35% saline solution; (**b**) cleaned insulator chain. Right-side perspective.

Cleaning tests were conducted on this insulator chain by manually flying the drone. A 35% saline solution was added to the glass disc insulators’ surface to enable a visual assessment of cleaning efficacy. For laboratory tests, a higher concentration of salt was deposited on the insulators’ surface than that found in practice. This was performed to ensure that the salinity present on field insulators could be effectively removed by the developed drone–robot. Figure 13, Figure 14, Figure 15 and Figure 16 show the discs covered with saline solution before and after cleaning.

**Figure 17:**
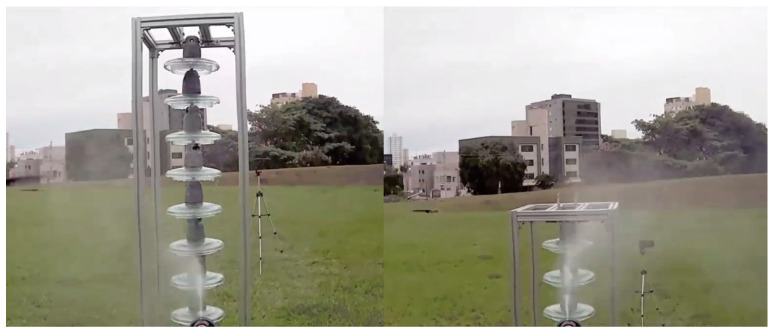
Washing of insulators viewed by the drone’s front camera with the depth camera’s distance control system.

In the cleaning tests, the drone–robot was manually approached by the chain and the washer jet was activated from the ground station. Water jet cleaning was performed frontally in relation to the chain, with vertical upward and downward movements of the drone–robot and side movements, as shown in Figure 17.

Although this paper has proven the effectiveness of cleaning chains polluted with salt, other pollutants can also be removed, according to Table 1, depending on their characteristics.

With the distance detection system using a depth camera, tests were conducted on the system’s effectiveness in maintaining a safe distance from the insulator chain. The auditory warning system was used to monitor the distance at which the drone was from the insulator chain. The depth camera image was displayed on a portable screen, enabling a visualization of when the chain was in an adequate position for cleaning.

Figure 17 presents a view from the drone–robot’s RGB camera, which helps the operator to position the flight and jet for more effective cleaning. Maintaining the safety distance in this test was accomplished using the developed algorithm with the depth camera.

### 3.2. Field Tests

**Figure 18:**
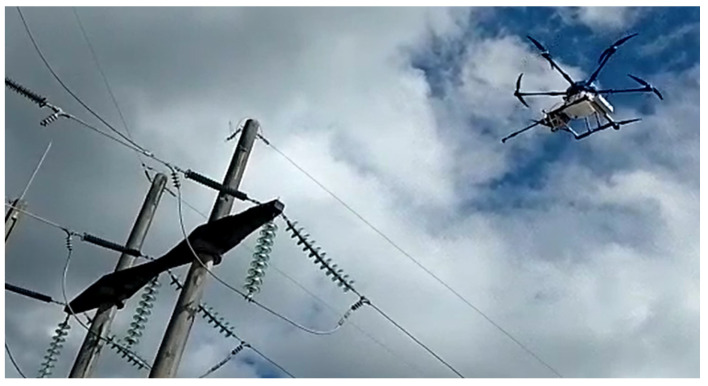
Drone–robot applied to an energized 138 kV insulator chain.

After the simulated tests, detailed in Section 3.1, tests were carried out on the high-voltage power lines of CELESC to verify the shielding system, the drone’s stability against electromagnetic interference, and the vision system for controlling the drone’s distance from the insulator chain. Washing tests on an insulator chain were performed at an electricity supply substation, where the insulators’ voltage was 138 kV. A vertical chain was used (Figure 18).

**Figure 19:**
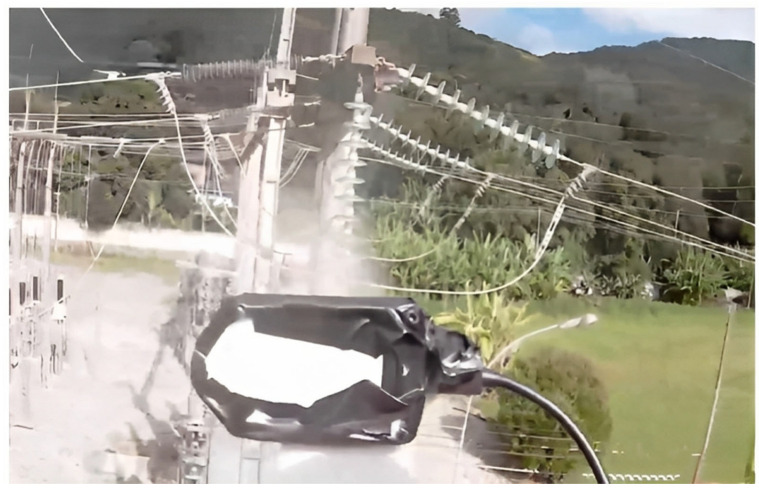
RGB camera view of the experimental test.

**Figure 20:**
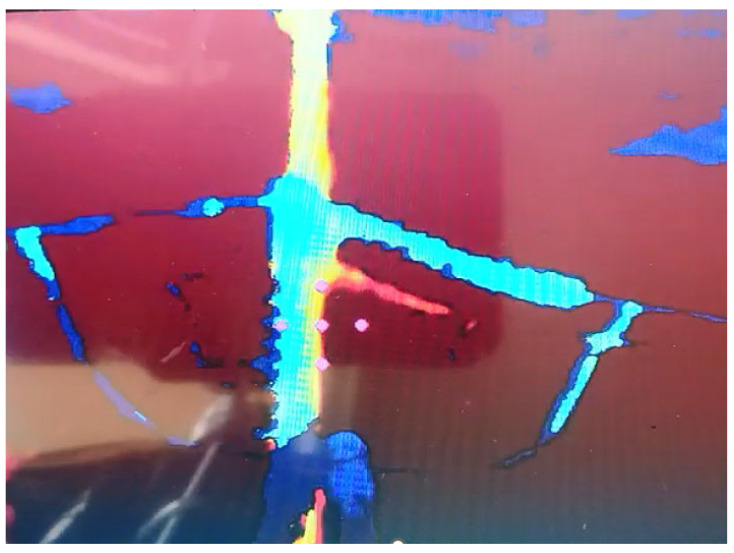
Depth vision system developed to maintain a safe distance from the insulator chain.

The drone’s reservoir was filled with 8 L of demineralized water (free of mineral salts, ions and other impurities, with very low conductivity and a maximum of 10 microsiemens and a pH of 7.1 to 7.4) in order to prevent the conduction of electricity from the insulators to the drone–robot. The operation was guided with the aid of the drone–robot’s RGB front camera (Figure 19) and the depth camera (Figure 20). The drone–robot was positioned by the operator in front of the chain to carry out the cleaning, respecting the safety distance.

During and after the tests, no electrical effect was noticed on the electronic system embedded in the drone–robot, and it was possible to turn the washer on and off, showing that the electronic system’s shielding was sufficient to prevent electromagnetic interference and electrostatic discharges. The electromagnetic interference did not interfere with the drone’s flight controller. The vision system using the depth camera enabled the drone to maintain a safe distance from the insulator chain (Figure 20).

## 4. Discussion

From Figure 13, Figure 14, Figure 15 and Figure 16, it can be inferred that the cleaning was successful, with a few spots of dirt that were not removed highlighted in red. It should be noted that these could be removed with subsequent cleanings after identification using the post-cleaning image acquisition of the insulators. Despite the cleaning being effective, it is necessary to automate the flight, with the ascent, descent, and lateral movements being performed by the vision system. The presented solution is dependent on the drone–robot operator’s skill.

Regarding field tests, the significant difficulties encountered for this cleaning operation with the proposed drone–robot were mainly related to the risk of collision risk during drone piloting or equipment failure, causing power supply interruption. Therefore, it is necessary to implement an automated control system during cleaning to enhance operational safety and reduce the risk of accidents.

The video of the experimental tests can be accessed at: https://drive.google.com/file/d/19NN_7l435U8hYKSPuIaArxOyxhIghUBv/view?usp=share_link (accessed on 08 June 2023).

## 5. Conclusions

The electric power transmission system requires daily maintenance. This is carried out by technicians and involves various risks to their lives. A critical point for power companies is the maintenance of insulator chains, which require periodic cleanings to prevent the possibility of line shutdown due to various pollutants.

In this paper, the possibility of cleaning insulator chains with the proposed drone–robot was proven, offering an agile, effective solution that reduces the chance of accidents with employees.

The developed solution’s limitations include the maximum flight time and the limited water reservoir capacity available for cleaning. These problems can be resolved with the provision of several batteries or quick chargers. Despite the reservoir’s limited capacity, it is easily refilled on the ground.

The vision system needs improvements to better integrate the RGB and the visualization of the depth camera that is available to the drone–robot operator.

It is essential for the drone–robot to be guided by a properly trained operator who is familiar with the system and has flight experience, thus preventing accidents and ensuring that cleaning is performed to the highest possible quality.

Future work proposes the automation of necessary cleaning procedures and the large-scale application of the solution.

## Figures and Tables

**Table 1:** Water washing method allowed according to the type of contaminant to be washed (Adapted from [16]).

Contaminant	Low-Pressure Washing	High-Pressure Washing
Salt	Yes	Yes
Cement/lime	No	No
Dirt dust	Yes	Yes
Fertilizer dust	Yes	Yes
Metallic dust	No	No
Coal dust	Yes	Yes
Volcanic ash	Yes	Yes
Bird droppings	Yes	Yes
Chemical	Yes	No
Vehicle smoke	Yes	Yes
Cooling tower effluent	Yes	Yes
Coal smoke/soot	Yes	Yes
Organic	Yes	Yes
Ice/snow	No	Yes
Oil/grease	No	No

**Table 2:** Specifications related to the drone–robot’s cleaning system for cleaning insulator chains.

Cleaning Method	Water Washing
Contaminants targeted by washing	Salt
Type of insulator to be cleaned	Glass or ceramic
Pressure	320 psi
Flow rate	1.6 L/min
Jet shape	Droplets/spray
Safety distance	1.5 m to 1.0 m from chain
Maximum wind speed	8 m/s

## Data Availability

All necessary data have been inserted in the article.

## Supplementary Materials

The following supporting information can be downloaded at: https://drive.google.com/file/d/19NN_7l435U8hYKSPuIaArxOyxhIghUBv/view?usp=share_link (accessed on 8 June 2023).
